# Wearable derived cardiovascular responses to stressors in free-living conditions

**DOI:** 10.1371/journal.pone.0285332

**Published:** 2023-06-02

**Authors:** David M. Presby, Summer R. Jasinski, Emily R. Capodilupo

**Affiliations:** Department of Data Science and Research, Whoop, Inc., Boston, Massachusetts, United States of America; Vels Institute of Science, Technology and Advanced Studies (VISTAS), MALAYSIA

## Abstract

Stress contributes to the progression of many diseases. Despite stress’ contribution towards disease, few methods for continuously measuring stress exist. We investigated if continuously measured cardiovascular signals from a wearable device can be used as markers of stress. Using wearable technology (WHOOP Inc, Boston, MA) that continuously measures and calculates heart rate (HR) and heart rate variability (root-mean-square of successive differences; HRV), we assessed duration and magnitude of deviations in HR and HRV around the time of a run (from 23665 runs) or high-stress work (from 8928 high-stress work events) in free-living conditions. HR and HRV were assessed only when participants were motionless (HR_motionless_). Runs were grouped into light, moderate, and vigorous runs to determine dose response relationships. When examining HR_motionless_ and HRV throughout the day, we found that these metrics display circadian rhythms; therefore, we normalized HR_motionless_ and HRV measures for each participant relative to the time of day. Relative to the period within 30 minutes leading up to a run, HR_motionless_ is elevated for up to 180–210 minutes following a moderate or vigorous run (P<0.05) and is unchanged or reduced following a light run. HRV is reduced for at least 300 minutes following a moderate or vigorous run (P<0.05) and is unchanged during a light run. Relative to the period within 30 minutes leading up to high-stress work, HR_motionless_ is elevated during and for up to 30 minutes following high-stress work. HRV tends to be lower during high-stress work (P = 0.06) and is significantly lower 90–300 minutes after the end of the activity (P<0.05). These results demonstrate that wearables can quantify stressful events, which may be used to provide feedback to help individuals manage stress.

## Introduction

Stress has been implicated as a contributing factor to numerous diseases, including cardiovascular disease, depression, and certain types of cancers [1, 2]. The number of stressful events, the degree to which someone responds to a stressful event, and the time it takes to recover from a stressful event have been found to be associated with mortality and poor mental and physical health [3, 4]. Despite the links between stress and disease, few methods exist that allow people to objectively quantify the amount of stress caused by an event; therefore, many individuals lack the knowledge to avoid or properly manage stressful events. To appropriately manage stress, methods that quantify the impact and duration of stressful events in real-time need to be developed.

In controlled laboratory settings, stress is typically gauged with surveys, cardiovascular measures, or blood/saliva tests. However, each of these methods are limited in their ability to be used for widespread use: surveys require conscientious feedback that may not be possible during or right after a stressful event; cardiovascular measures are classically acquired using leads for electrocardiograms and sphygmomanometers for measuring blood pressure, which require bulky equipment and specialized knowledge to use; and blood or saliva tests require specialized equipment and trained technicians to measure the concentration of stress hormones. Translating these laboratory-grade stress examinations into tests that are easier to perform and interpret may promote greater adoption of stress tracking.

Certain wearable devices continuously measure cardiovascular parameters using photoplethysmography and provide a unique opportunity to investigate the magnitude of stressful events on cardiovascular parameters in real-time. In particular, HR and HRV have the potential to be surrogate markers of stress, whereby both HR or HRV can vary in response to the degree of sympathetic or parasympathetic input [5–7]. Recent research has investigated the ability of wearable-derived metrics to detect or measure stressful events, like infections and vaccines, when aggregated over a long periods of time [8–10]. In this study, we aimed to leverage the continuous monitoring provided by the WHOOP strap (WHOOP Inc, Boston, MA) to determine the magnitude of stressful events on cardiovascular metrics in free-living conditions.

## Methods

### Data collection

Since data were not identifiable and were stored on a secure server, this study was deemed exempt from Institutional Review Board (IRB) oversight by Advarra’s IRB (Columbia, MD). Participants were excluded from the study if they were under the age of 21 and if they indicated a gender other than male or female. A wrist-worn device (WHOOP versions 3.0 and 4.0; Boston, MA) that continuously collects heart rate, three-axis accelerometer, temperature, and three-axis gyroscope data was used to calculate cardiovascular metrics and sleep and wake times, which have been validated elsewhere [11]. Running and high-stress work activities are logged via manual input from a participant or auto-detected by the WHOOP analytics platform. Sleep and wake onset are autodetected. To be considered for this analysis, a user must have had at least 25 days of data over a 28-day span and no more than 28 days of data were used for each participant.

### Data calculation

#### Resting heart rate and heart rate variability

Heart rate (HR_motionless_) and heart rate variability (HRV) were calculated during periods that lacked motion detected by the accelerometer. Moving-block sub-sampling was used to calculate HR_motionless_ and HRV. The step size was 30 seconds and utilized a 5-minute block of photoplethysmography-derived beat-to-beat intervals and accelerometry data to calculate HR_motionless_ and HRV in the absence of motion artifacts. Heart rate variability was calculated as the root-mean-square of successive differences. HR_motionless_ and HRV derived from the WHOOP device have been validated against gold-standard measures from electrocardiograms [11].

#### Relative fractional differences

To calculate the relative fractional difference for resting heart rate and heart rate variability throughout the day, we first calculated the median resting heart rate and heart rate variability for each hour of the day over a 28-day period for each participant. We then divided the median daily HR_motionless_ and HRV by each hour’s median HR_motionless_ and HRV value to determine the relative fractional difference of each hour’s HR_motionless_ and HRV from the median daily HR_motionless_ and HRV within each user.

#### Impact of an activity

HR_motionless_ and HRV were aggregated over the duration of the activity or over 30-minute intervals before the start of or following an activity. Aggregated HR_motionless_ and HRVs were then subtracted by the median hourly HR_motionless_ and HRV for that time of day for a given user. Lastly, we subtract the post-activity normalized HR_motionless_ and HRV values from the pre-activity normalized HR_motionless_ and HRV values. Cardiovascular parameters were not calculated during the running activity due to motion artifacts which prevent the reliable acquisition of the photoplethysmography data [12]. To determine whether a running load was light, moderate, or vigorous, we utilized a proprietary algorithm that scores a run using the amount of time spent in a cardiovascular zone based on an individual’s heart rate reserve.

#### Statistical analysis

Data analyses were conducted in either Python (version 3.8.12) or R (version 4.2.0). Descriptive statistics are provided as frequencies and percentages for categorical variables or means and 95% confidence intervals for continuous variables. To assess circadian rhythms in HR_motionless_ and HRV, we fit splines to linear models to determine the non-linear relationships. To determine if HR_motionless_ and HRV deviated from baseline relative to the period before a run or high-stress work, we first modeled the data using linear mixed-effect models and then utilized Dunnet’s test as a post-hoc test that directly compared the time intervals following an activity to the time before an activity. To determine the difference in HR_motionless_ and HRV between running loads, we calculated areas under the curve using the trapezoidal method [13] (treating 0 as the baseline) for each running load group and compared these areas under the curve using Tukey’s test. Significance was set as P<0.05.

## Results

### Diurnal fluctuations in HR_motionless_ and HRV

To better understand the diurnal variation that occurs throughout the day, we analyzed normalized hour-to-hour fluctuations in HR_motionless_ and HRV throughout the day for 974 individuals (Table 1) aggregated within a given clock hour or hours to and from sleep or wake.

**Table 1:** Demographics of participants for circadian analysis.

	Overall (N = 974)
**Gender**	
female	273 (28.0%)
male	701 (72.0%)
**Age**	
Mean (CI)	36.849 (36.196, 37.503)
**Height**	
Mean (CI)	1.766 (1.760, 1.772)
**Weight**	
Mean (CI)	80.850 (79.848, 81.852)
**BMI**	
Mean (CI)	25.818 (25.560, 26.075)

**Fig 1:**
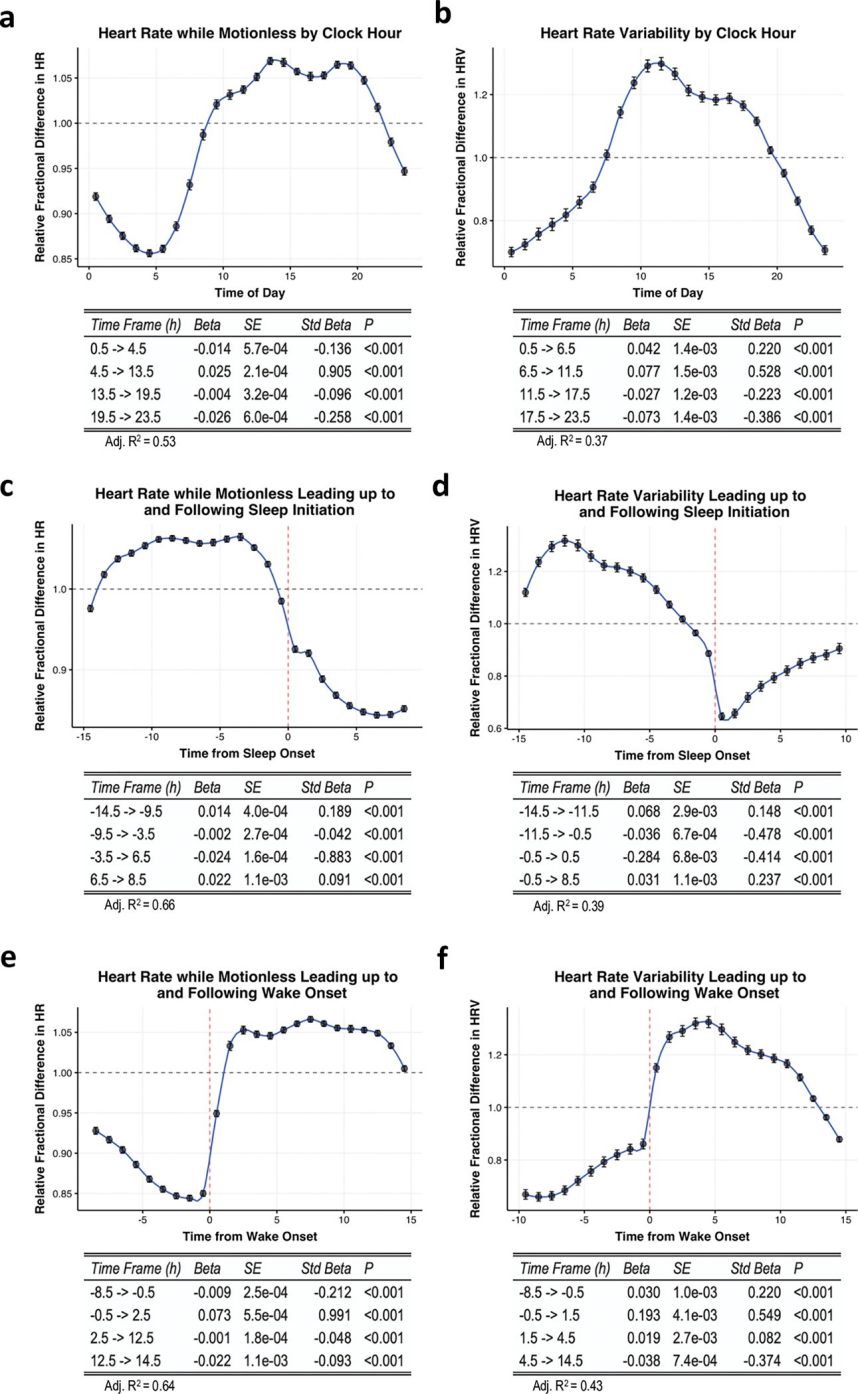
Diurnal variations in HR_motionless_ and HRV. Data are aggregated for each hour over 28 days for each participant and expressed as the relative fractional difference from the average throughout the day. (**a)** HR_motionless_ relative to time of day. (**b)** HRV relative to time of day. (**c)** HR_motionless_ leading up to and following sleep onset. (**d)** HRV leading up to and following sleep onset. (**e)** HR_motionless_ leading up to and following wake onset. (**f)** HRV leading up to and following wake onset. Tables below each figure represent the results from the linear spline models. Data are represented by means ± 95% CI.

Within a given clock hour (Fig 1A and 1B), HR_motionless_ decreases from time point 0.5 to 4.5 (β = -0.014, P<0.001), increases from time point 4.5 to 13.5 (β = 0.025, P<0.001), negligibly decreases from time point 13.5 to 19.5 (β = -0.004, P<0.001), and decreases from time point 19.5 to 23.5 (β = -0.026, P<0.001). For HRV within a given clock hour (Fig 1B), we find that HRV increases from time point 0.5 to 6.5 (β = 0.042, P<0.001) and time point 6.5 to 11.5 (β = 0.077, P<0.001), and decreases from time point 11.5 to 17.5 (β = -0.027, P<0.001) and 17.5 to 23.5 (β = -0.073, P<0.001).

We next modeled fluctuations in HR_motionless_ and HRV leading up to and following sleep, with negative numbers reflecting hours until sleep onset and positive numbers reflecting hours after sleep (Fig 1C and 1D). HR_motionless_ increases from time point -14.5 to -9.5 (β = 0.014, P<0.001), negligibly decreases from -9.5 to -3.5 (β = -0.002, P<0.001), decreases from -3.5 to 6.5 (β = -0.024, P<0.001), and negligibly increases from 6.5 to 8.5 (β = 0.022, P<0.001). HRV increases from time point -14.5 to -11.5 (β = 0.068, P<0.001), decreases from -11.5 to -0.5 (β = -0.036, P<0.001) and -0.5 to 0.5 (β = -0.284, P<0.001), and increases from 0.5 to 8.5 (β = 0.031, P<0.001).

Lastly, we modeled fluctuations in HR_motionless_ and HRV leading up to and following wake, with negative numbers representing hours until wake and positive numbers representing hours after wake (Fig 1E and 1F). HR_motionless_ decreases from time point -8.5 to -0.5 (β = -0.009, P<0.001), increases from -0.5 to 2.5 (β = 0.073, P<0.001), and negligibly decreases from 2.5 to 12.5 (β = -0.001, P<0.001) and from 12.5 to 14.5 (β = -0.022, P<0.001). HRV increases from time point -8.5 to -0.5 (β = 0.03, P<0.001) and from -0.5 to 1.5 (β = 0.193, P<0.001), negligibly increases from 1.5 to 4.5 (β = 0.019, P<0.001), and decreases from 4.5 to 14.5 (β = -0.038, P<0.001).

### Direct and residual effects of physical and psychological stressors on HR_motionless_ and HRV

**Fig 2:**
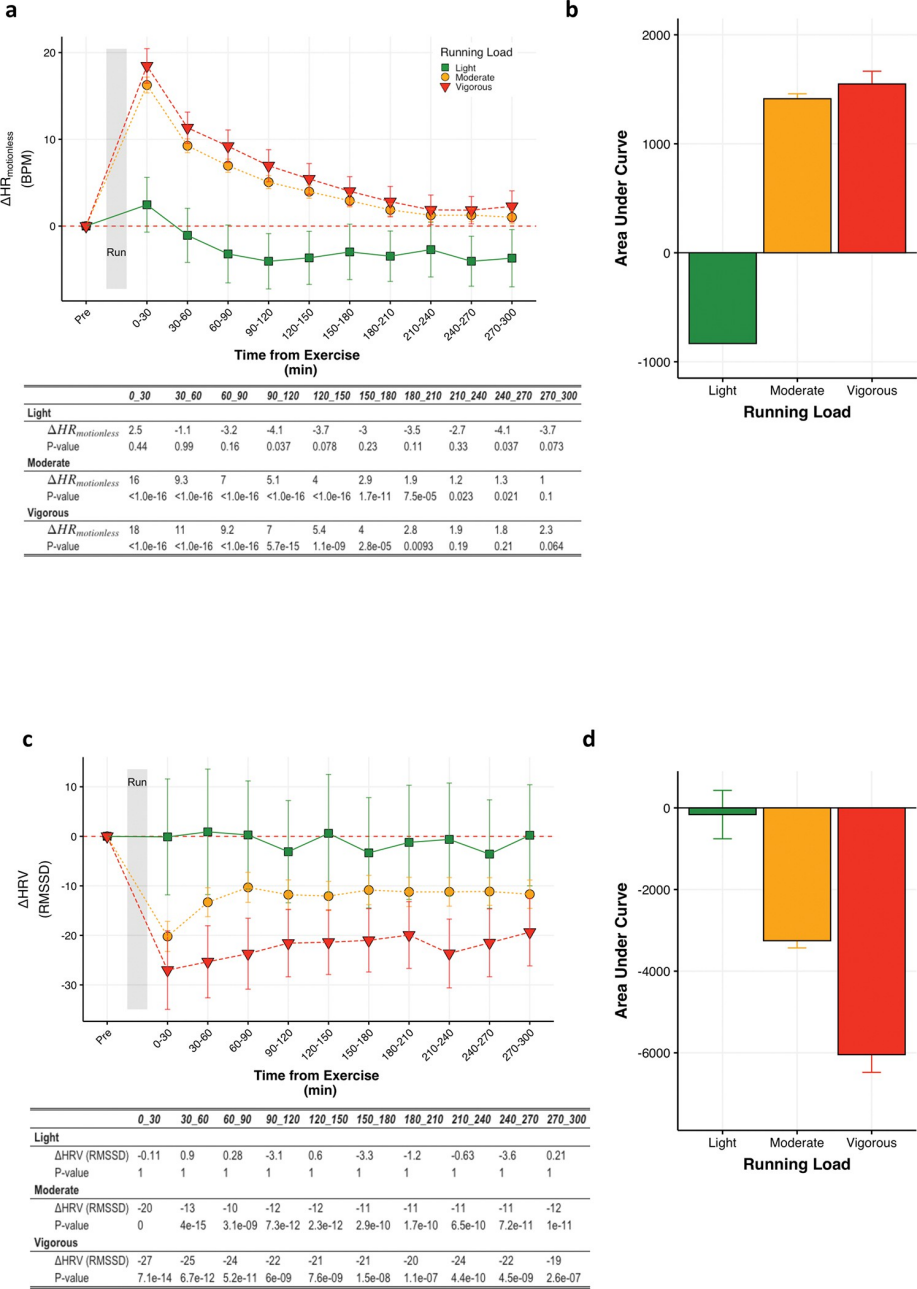
Dose-response effects of running on HR_motionless_ and HRV. Data are aggregated over 30 minute blocks of time leading up to and following a run, and are normalized to time of day and the thirty minutes leading up to the run (“Pre”). (**a)** Change in HR_motionless_ (BPM) relative to the period before a run. The table reflects the absolute differences and P-values for HR_motionless_ following a run, grouped by running load. P-values were determined using Dunnet’s test, treating the period before a run (“Pre”) as the control group. **(b)** Area under the curve for HR_motionless_ following a run. P-values were determined using Tukey’s test with comparisons made across all groups. (**c)** Change in HRV (RMSSD) relative to the period before a run. The table reflects the absolute differences and P-values for HRV following a run, grouped by running load. P-values were determined using Dunnet’s test, treating the period before a run (“Pre”) as the control group. **(d)** Area under the curve for HRV following a run. P-values were determined using Tukey’s test with comparisons made across all groups. Data are represented by means ± 95% CI.

To characterize the effects of a physical stressor on cardiovascular parameters, we examined HR_motionless_ and HRV starting from 30 minutes leading up to and 5 hours following 23665 running activities (Table 2). Furthermore, we aimed to determine if we could detect a dose effect of running on HR_motionless_ and HRV and grouped runs into light, moderate, and vigorous loads based on time spent in cardiovascular zones. Relative to the period before the run, HR_motionless_ remains elevated after a moderate or vigorous run for at least 180–210 minutes (P<0.05) and is unchanged or decreases after a light run (Fig 2A). Using the area under the curve to compare the relative change in HR_motionless_ post-run between running loads we find that, compared to light runs, both moderate and vigorous runs led to greater elevations in HR_motionless_ (P<0.05; Fig 2B). Relative to the period before the run, HRV remains depressed after a moderate and vigorous run for at least 270–300 minutes (P<0.05) and is unchanged after a light run (Fig 2C). Using the area under the curve to compare the relative change in HRV post-run between running loads, we find that vigorous runs lead to larger decreases in HRV than moderate to vigorous runs (P<0.05; Fig 2D).

**Table 2:** Demographics of running populations and descriptive statistics of associated runs.

	Light (N = 463)	Moderate (N = 1834)	Vigorous (N = 1097)
**Gender**			
Female	221 (47.7%)	833 (45.4%)	502 (45.8%)
Male	242 (52.3%)	1001 (54.6%)	595 (54.2%)
**Age**			
Mean (CI)	37.404 (36.414, 38.394)	36.437 (35.969, 36.906)	35.486 (34.893, 36.079)
**Height (m)**			
Mean (CI)	1.732 (1.723, 1.742)	1.734 (1.730, 1.739)	1.732 (1.726, 1.737)
**Weight (kg)**			
Mean (CI)	74.983 (73.462, 76.505)	73.834 (73.166, 74.501)	72.237 (71.443, 73.032)
**BMI**			
Mean (CI)	24.793 (24.420, 25.167)	24.402 (24.241, 24.564)	23.954 (23.765, 24.142)
**Number of Runs**			
Count	1640.000	17085.000	4940.000
**Number of Runs Logged per Person**			
Mean (CI)	3.542 (3.098, 3.986)	9.316 (9.029, 9.602)	4.503 (4.245, 4.761)
**Max Heart Rate (BPM)**			
Mean (CI)	146.536 (144.519, 148.552)	174.751 (174.330, 175.171)	183.068 (182.567, 183.569)
**Average Heart Rate (BPM)**			
Mean (CI)	116.283 (114.278, 118.288)	144.803 (144.321, 145.284)	154.626 (154.072, 155.180)
**Duration (min)**			
Mean (CI)	26.239 (21.662, 30.816)	43.746 (42.975, 44.516)	83.818 (80.490, 87.146)

Heart rate includes measures taken in the presence of motion.

**Fig 3:**
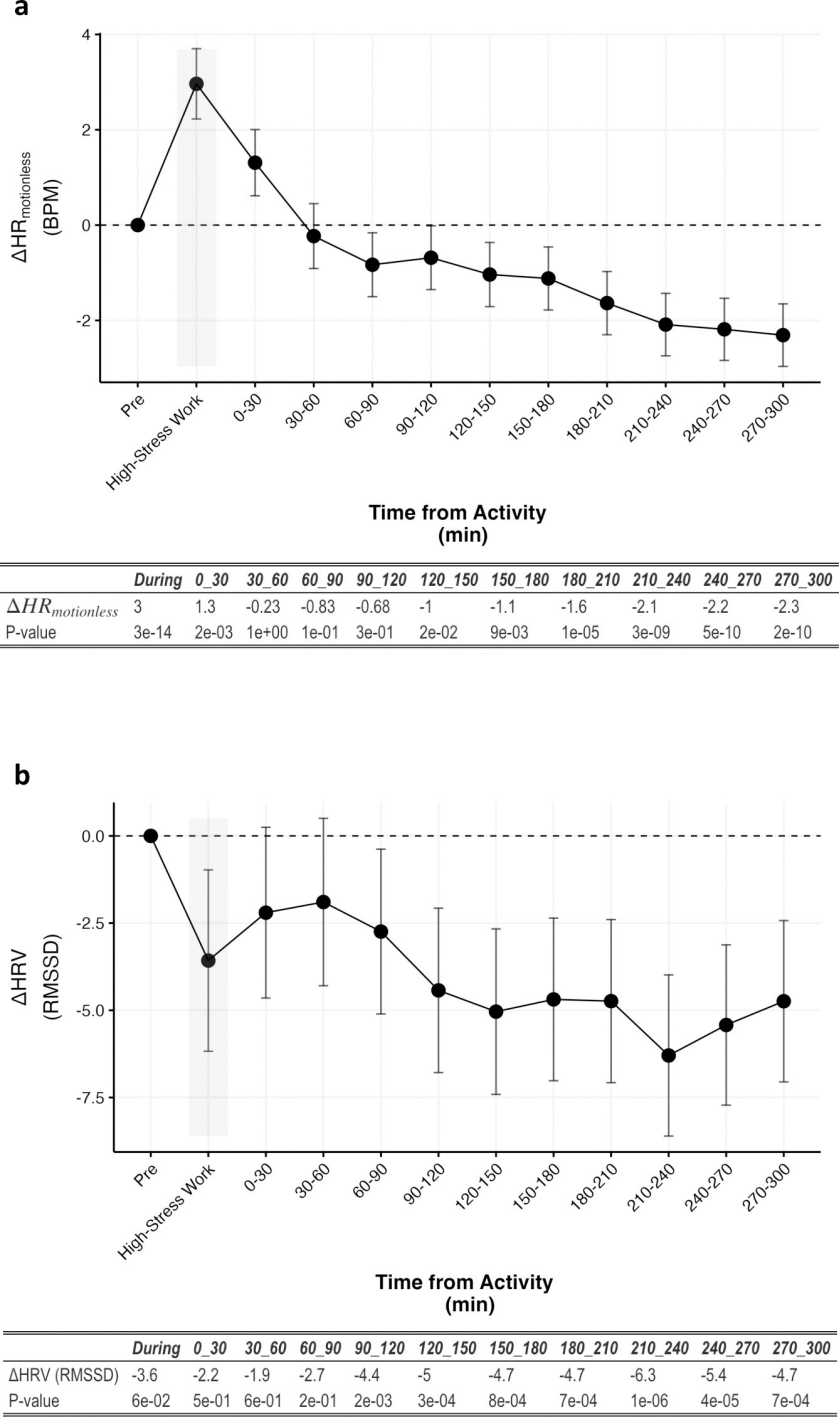
Effects of high-stress work on HR_motionless_ and HRV. Data are aggregated over 30 minute blocks of time leading up to, during, and following high-stress work, and are normalized to time of day and the thirty minutes leading up to the high-stress work. (**a)** Change in HR_motionless_ (BPM) relative to the period before high-stress work. (**b)** Change in HRV (RMSSD) relative to the period before high-stress work. The table reflects the absolute differences and P-values for HR_motionless_ or HRV following a high-stress work. P-values were determined using Dunnet’s test, treating the period before a high-stress work (“Pre”) as the control group. Data are represented by means ± 95% CI.

To characterize the effects of a psychological stress on cardiovascular parameters, we examined HR_motionless_ and HRV starting from 30 minutes leading up to and 300 minutes following 8928 high-stress work periods (Table 3). We find that HR_motionless_ is elevated during and for up to 30-minutes after high-stress work (P<0.05; Fig 3A) and after thirty-minutes is either unchanged or depressed (P>0.05; Fig 3A). HRV trended towards being lower during high-stress work (P = 0.06; Fig 3B) and was significantly lower 90–300 minutes after the event (P<0.05; Fig 3B). In summary, these results suggest that HR_motionless_ and HRV from wearables may be used as surrogate markers of physical or psychologically stressful events.

**Table 3:** Demographics of high-stress work population and descriptive statistics of associated high-stress work events.

	Overall (N = 1778)
**Gender**	
Female	536 (30.1%)
Male	1242 (69.9%)
**Age**	
Mean (CI)	36.916 (36.339, 37.493)
**Height (m)**	
Mean (CI)	1.756 (1.751, 1.760)
**Weight (kg)**	
Mean (CI)	80.338 (79.527, 81.148)
**BMI**	
Mean (CI)	25.916 (25.704, 26.127)
**Number of High-Stress Works**	
Count	8928.000
**Number of High-Stress** **Works Logged per Person**	
Mean (CI)	5.021 (4.625, 5.418)
**Max Heart Rate (BPM)**	
Mean (CI)	149.561 (148.576, 150.545)
**Average Heart Rate (BPM)**	
Mean (CI)	113.630 (112.586, 114.674)
**Duration (min)**	
Mean (CI)	114.792 (108.945, 120.638)

Heart rate includes measures taken in the presence of motion.

## Discussion

The response to a stressor can be measured via reactivity–how much the system deviates from baseline–and recovery–how long it takes the system to return to baseline [14]–and both reactivity and recovery can be quantified using cardiovascular parameters [15]. Improving awareness into how the body responds to stress, termed interoception, may lead to healthier stress responses [16]; therefore, easily-accessible methods to quantify the cardiovascular impact of stressors, which may improve interoception, may help individuals manage their stress. In this investigation, we collated self-reported physically and psychologically stressful events with objective measures from a wearable that continuously measures cardiovascular parameters (WHOOP Inc, Boston, MA). We find that individuals wearing WHOOP display circadian rhythms in their HR_motionless_ and HRV, and that deviations from an individual’s circadian rhythm can be used to gauge the magnitude of a stressful event based on HR_motionless_ and HRV.

A key finding from this study is that HR_motionless_ and HRV exhibit a circadian rhythm that aligns with the time a person falls asleep or wakes up. This finding is novel as this study is the first to investigate patterns of HR_motionless_ and HRV relative to when someone falls asleep or wakes up. Using clock time to assess circadian rhythms, prior research indicates that HR is elevated during day time and decreases leading up to and throughout the night, whereas HRV increases throughout sleep, peaks in the early morning, and subsequently decreases throughout the day [17, 18]. Since HR and HRV exhibit circadian rhythms, measures that rely on these cardiovascular parameters may need to be corrected for the time of day when the measure was taken.

Correcting for the time of day when HR_motionless_ and HRV were measured, we sought to determine how different types of stressors might impact cardiovascular dynamics in real-time conditions. High-intensity physical stressors, like exercise, can elevate HR and reduce HRV for many hours after the physical stressor has been removed due to persistent activation of the sympathetic nervous system and inhibition of the parasympathetic nervous system [19, 20]. Conversely, low-intensity exercise has been shown to reduce heart rate for up to 24 hours following the exercise bout, possibly due to an acute reduction in total and regional vascular resistance [21, 22]. We find that we can replicate these laboratory-based findings in real-world conditions by examining the impact of different running loads on cardiovascular dynamics post-exercise. These findings indicate that data derived from wearable devices can be used to assess the impact of an exercise session on the cardiovascular system, which may be useful in determining the efficacy of the exercise and assessing short-term recovery from exercise. Moreover, these results may also have health implications as the rate of cardiovascular recovery from exercise has been shown to be a predictor of mortality [23].

We also investigated how cardiovascular dynamics change in response to self-perceived high-stress work, which may consist of a psychological stress, physical stress, or a combination of the two. Psychological stress impacts cardiovascular metrics in a similar manner as physical stress—by increasing HR and reducing HRV during and following a stressful event [24, 25]. In this investigation, we find that HR_motionless_ is elevated during and immediately after high-stress work, whereas HRV tends to be reduced during and following 90 minutes after high-stress work. One plausible explanation for the 90-minute lag observed with a stressor-induced reduction in HRV following high-stress work is that this lagged-response may be due to an immediate relief of the stressor being removed (i.e., HRV returning to near pre-stressor levels) followed by a rumination period [26]; however, this hypothesis is only speculative and requires further research to validate.

In summary, the results from this study indicate that HR_motionless_ and HRV exhibit a circadian rhythm that can be used as a baseline to detect deviations in HR_motionless_ and HRV caused by stressful events. These results have practical applications as they demonstrate a novel method of normalization to detect deviations in stress-related metrics and provide evidence that detecting stress can be done passively and outside of clinical settings. An interesting future direction for research will be to assess if these wearable-derived cardiovascular markers of stress can predict the onset of certain diseases [27]. Ultimately, these findings may be useful in detecting stressful events, increasing interoception, and improving responses to stressors.
